# Evaluation of Synthetic GnRH-Analog Peforelin with Regard to Oocyte Differentiation and Follicular Development in C57BL/6J Mice

**DOI:** 10.3390/ani14192866

**Published:** 2024-10-04

**Authors:** Lena Amberger, Daniel Wagner, Sonja Höflinger, Frederik Zwicker, Dana Matzek, Bastian Popper

**Affiliations:** Biomedical Center, Core Facility Animal Models, Faculty of Medicine, Ludwig-Maximilians-Universität München, Großhaderner Straße 9, 82152 Planegg-Martinsried, Germany

**Keywords:** gonadotropin, Maprelin, superovulation, IVF

## Abstract

**Simple Summary:**

This study evaluated peforelin as a potential alternative to PMSG for the induction of superovulation in C57BL/6J mice. The primary outcomes measured included oocyte yield, fertilization rates, and ovarian morphology. The results showed that although peforelin was less effective than PMSG in stimulating oocyte development and follicle differentiation, it did not adversely affect oocyte quality or ability to reach the blastocyst stage after in vitro fertilization. This study concludes that peforelin, particularly at 0.5 µg, is a promising alternative to PMSG, although further research is needed to optimize its use in the laboratory. This research supports the development of alternatives to PMSG in line with ethical animal welfare considerations.

**Abstract:**

In biomedical research, ovulation induction is a critical step in the reproductive biology of laboratory animals. This study evaluates the efficacy of peforelin, a synthetic gonadotropin-releasing hormone (GnRH) analog, in comparison to pregnant mare serum gonadotropin (PMSG, synonym: eCG), traditionally used for ovulation induction in mice. PMSG is derived from the serum of pregnant horses, and its production is becoming increasingly problematic due to animal welfare concerns and regulatory restrictions. The aim of this study was, therefore, to evaluate an ethically acceptable and less invasive alternative to PMSG. Female C57BL/6J mice, aged 3–4 weeks, were divided into two groups to receive either peforelin at three different concentrations or PMSG, followed by an injection of human chorionic gonadotropin (hCG) to induce ovulation. Key outcomes included the number and quality of oocytes collected, fertilization rates, ovary morphology, and follicular differentiation. Although the number of oocytes was significantly lower in the peforelin cohort, the fertilization rate was high. Ovarian morphology was not significantly altered compared to the PMSG cohort. This study showed that peforelin is suitable for superovulation in mice. These results suggest that peforelin could be an ethically acceptable alternative to PMSG stimulation for inducing superovulation in mice.

## 1. Introduction

Ovulation induction is a fundamental process in reproductive research and is widely used in the development of genetic models and pharmacological testing. The conventionally used pregnant mare serum gonadotropin (PMSG), also known as equine chorionic gonadotropin (eCG), is extracted from the serum of pregnant horses and is widely used due to its high efficacy in inducing ovulation in various mammals [1,2,3,4]. However, PMSG extraction methods raise significant ethical concerns, as they require invasive procedures on pregnant horses [5]. Moreover, significant scientific and public debate surrounding hormone extraction methods necessitates the exploration of alternative methods. PMSG/eCG mimics the endogenous hormone follicle-stimulating hormone (FSH), which is secreted by cells of the pituitary gland and stimulates the maturation of follicles in the ovary [6]. The release of FSH is regulated by releasing hormones secreted by neurons in the hypothalamus that exert their effect on pituitary cells [7].

The utilization of recombinant hormones, which are generated using biotechnological methods, presents a promising alternative to conventional methods of hormone extraction from animal-derived sources. Recent work has shown that recombinant GnRH, FSH, and LH are being used as therapeutic options in both veterinary and human medicine, while recombinant eCG is primarily utilized in veterinary applications [8,9,10,11]. Their controlled synthesis allows for high purity and specific biological activity [12], which is particularly important for the induction of ovulation and the treatment of reproductive disorders. Recombinant GnRH generally mimics the natural form of the hormone and has a relatively short half-life, similar to endogenous GnRH. This short half-life is due to rapid degradation by peptidases, which limits sustained action. Despite that, synthetic and therefore structurally modified GnRH derivates provide more precise control over hormonal surges and declines, making them advantageous in certain clinical applications [13].

In this respect, peforelin (Maprelin^®^, Veyx–Pharma GmbH, Schwarzenborn, Germany), a synthetic analog of gonadotropin-releasing hormone (GnRH), offers a potential alternative that could eliminate the ethical burden associated with the use of animal-derived PMSG/eCG products. Previous studies have documented the efficacy of peforelin in pigs [14,15], but its use in ovulation induction in laboratory mice, particularly in the widely used C57BL/6J strain, has not yet been investigated.

This research gap highlights a significant potential for ethical improvements in biomedical research with respect to the 3R principle [16]. The aim of this study is to evaluate the efficacy of three different doses of peforelin compared to standard protocols using PMSG in inducing superovulation in C57BL/6J mice. Primary endpoints include oocyte number and quality, as well as oocyte differentiation and fertilization rates. Furthermore, we investigated follicular stages and corpora lutea in histological sections of dissected ovaries. The present work aims to establish peforelin as an effective and ethical alternative to PMSG by evaluating its effects on ovulation induction and related physiological processes in mice. Our findings could not only have important implications for improving animal welfare but also provide a basis for advancing protocols in reproductive research and animal biotechnology.

## 2. Materials and Methods

This trial was approved by the government of Upper Bavaria (Az. 02-22-078). Housing of laboratory mice was in accordance with European and German animal welfare legislations (5.1-231 5682/LMU/BMC/CAM). Four-week-old female C57BL/6J mice (Charles River, Sulzfeld, Germany) were housed in groups of four individuals per cage. Room temperature and relative humidity ranged from 20 to 22 °C and 45 to 55%. The light cycle was adjusted to 12 h light–12 h dark. Room air was exchanged 11 times per hour and filtered with HEPA systems. All mice were housed in individually ventilated cages (Typ II long, Tecniplast, Hohenpeißenberg, Germany) under specified pathogen-free conditions. Hygiene monitoring was performed every three months based on the recommendations of the FELASA-14 working group. All animals had free access to water and food (irradiated, 10 mm pellet; 1314P, Altromin, Lage, Germany). The cages were equipped with nesting material (5 × 5 cm, Nestlet, Datesand, Stockport, UK), a red corner house (Tecniplast, Hohenpeißenberg, Germany), and a rodent play tunnel (7.5 × 3.0 cm, Datesand, Stockport, UK). Soiled bedding (Animal bedding fine, LTE-E-002, Abedd, Vienna, Austria) was replaced every 7 days.

### 2.1. Hormonal Treatment

Mice were randomly divided into two groups to receive either three different concentrations of peforelin (trials 1–3, n = 10, respectively) or PMSG (trials 1–3, n = 2, respectively). Peforelin (Maprelin^®^, Veyx-Pharma GmbH, Schwarzenborn, Germany) was administered at doses of 1.0 µg, 0.5 µg, and 0.25 µg, while PMSG (Ceva Tiergesundheit GmbH, Düsseldorf, Germany) was given at a dose of 5 IU per mouse in all trials. All hormones were injected interperitoneally. Forty-eight hours after the hormonal treatment, all mice received an intraperitoneal injection of human chorionic gonadotropin (hCG) (Ovogest 300, MSD Tiergesundheit, Unterschleißheim, Germany) at 5 IU to induce ovulation.

### 2.2. Oocyte Collection and Analysis

**Figure 1:**
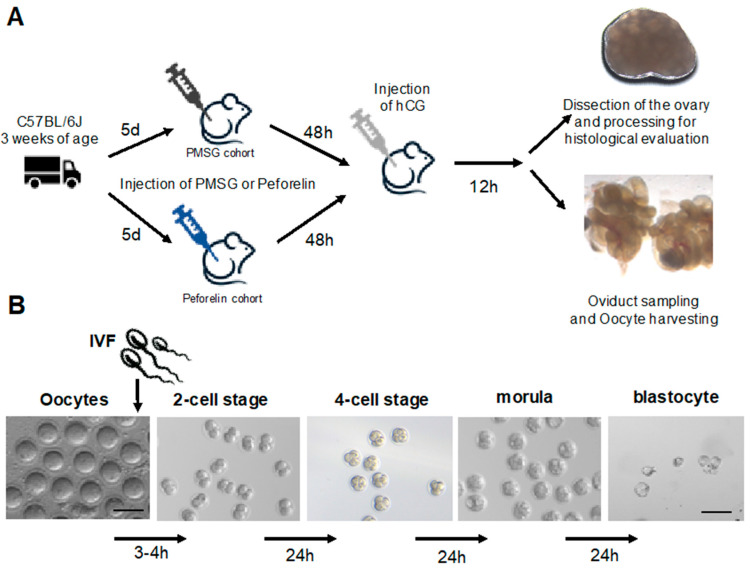
Workflow. PMSG and peforelin administration regime and sample preparation (**A**) and oocyte differentiation after in vitro fertilization (IVF) up to the blastocyte stage (**B**).

Oocytes were collected 12 h after the hCG treatment. Mice were euthanized by cervical dislocation, and oviducts and ovaries were immediately dissected (Figure 1), collected, and transferred to a fertilization dish covered with paraffin oil. Based on standard procedures, we pooled the oocytes per group and subjected them to further analysis [17]. Under microscopic observation, cumulus–oocyte complexes were collected from the oviducts and transferred to 90 µL of fertilization medium. The number of ovulated oocytes and the fertilization ability of oocytes in each group were examined. Furthermore, oocytes were counted under a light microscope (Olympus SZX10, Hamburg, Germany), and their quality was assessed based on the integrity of the zona pellucida and lysis of the cytoplasm. Further, blastocytes were evaluated with respect to the integrity of the blastocyte cavity.

### 2.3. Sperm Freezing and Thawing

After euthanasia of male C57BL/6J mice through cervical dislocation, their cauda epididymides were removed and completely cleansed of all fat and blood under a microscope. Subsequently, two small incisions were made using micro-spring scissors to allow the sperm to swim out. Freezing straws (0.25 mL plastic straw; Minitüb GmbH, Tiefenbach, Germany) were loaded with 100 µL M2 (Merck KGaA, Millipore, Darmstadt, Germany) and 10 µL sperm suspension, and both sides of the straw were heat-sealed. The sealed straws were cooled in the liquid nitrogen gas layer for 10 min. The straws were plunged directly into liquid nitrogen and stored until use.

### 2.4. In Vitro Fertilization

A straw containing cryopreserved sperm was removed from liquid nitrogen and thawed in a 37 °C water bath for 10 min. The thawed sperm suspension (10 µL) was added to 90 µL of sperm preincubation medium (Fertiup, Cosmobio, Tokyo, Japan) covered with paraffin oil (NidaCon International AB, Mölndal, Sweden). The thawed sperm were preincubated for 30 min at 37 °C in an atmosphere containing 5% CO_2_ to induce capacitation. Subsequently, the sperm suspension (10 µL) was carefully collected, transferred to 90 µL of HTF+GSH medium (Merck KGaA, Millipore, Darmstadt, Germany) containing cumulus–oocyte complexes (female mouse/drop), and cultured for 3–4 h at 37 °C in an atmosphere containing 5% CO_2_. At 3 h after insemination, the oocytes were washed using four drops of HTF (Merck KGaA, Millipore, Darmstadt, Germany) (90 µL), and the number of ovulated oocytes was counted. At 24 h after insemination, the rate of fertilization was calculated as the total number of two-cell embryos divided by the total number of inseminated oocytes and multiplied by 100. In embryo culture, a number of the four-cell embryos were transferred to 90 µL of KSOM (Merck KGaA, Millipore, Darmstadt, Germany) and incubated for 72 h. The fertilization rate was calculated as the total number of oocytes divided by the total number of two-cell embryos and multiplied by 100. Images were acquired using a SZX10 (Olympus, Hamburg, Germany) and/or a Stemi 508 microscope (Leica, Wetzlar, Germany).

### 2.5. Histological Analysis

Harvested ovaries were measured immediately after dissection in an unfixed state, and the volume of the ovaries was determined as follows.

volume = (length × width × width)/2

Ovaries were fixed in formalin, embedded in paraffin, sectioned, and stained with Hematoxylin and Eosin (H&E). Six animals were evaluated for the PMSG cohort and the peforelin cohort trials 1–3. Three sections, each 5 µm thick and 25 µm apart from each other, were used to quantify the number of follicular stages and corpora lutea. Follicles were classified as primary, secondary, and tertiary follicles. Atretic follicles as well as corpora lutea were quantified as well. For the definitions of morphological criteria, see Table 1. Periodic acid schiff (PAS) staining was applied to evaluate the intactness of the zona pellucida (ZP). Four animals were used to quantify deficit ZP in the PMSG and peforelin cohort. Images were acquired using a Axiovert 5 microscope connected to a Axiocam 205 camera system (Zeiss, Jena, Germany).

### 2.6. Statistics

Statistics were conducted using the prism GraphPad 5.04 (GraphPad Software, San Diego, CA, USA). Student’s *t*-test was applied for parametric data. The Kruskal–Wallis (K–W) test, followed by Dunn’s multiple comparison (non-parametric data), was used to determine the *p*-values. Data are shown as mean and SEM values if not stated otherwise. The significance level was set to *α* < 0.05.

## 3. Results

Peforelin was used to induce ovulation in three runs, each with a different concentration. Table 2 shows the evaluation of peforelin against the respective control group with PMSG at a standard concentration. In all trials in which we injected peforelin (trials 1–3), fewer single oocytes were obtained from a total of 10 animals treated with the hormone compared to only 2 animals in the PMSG cohort. Within the peforelin runs, the highest cell numbers were obtained in the 0.50 µg group (trial 2). A defined amount of cryopreserved mouse sperm was added to the oocytes so that the resulting cells could also be tested for fertility. The differentiation of two cells (2C) into a blastocyst (Bl) was always examined in comparison with PMSG. There were no differences in the division intervals, but the numbers within the two hormones differed. In trials 1–3, despite the low oocyte yield, good fertility numbers and differentiation up to the blastocyst were recorded despite the low yield of oocytes. In trials 1 and 2, hardly any cells were lost during in vitro fertilization (IVF), whereas the post-fertilization yield was lower in the PMSG group.

In trials 1–3, good fertility numbers and differentiation up to the blastocyst were recorded despite the low yield of oocytes (Table 2). Taking into account that the same sperm was used in peforelin and PMSG cohorts, hardly any cells were lost during IVF in trials 1 and 2, whereas the yields after fertilization were poorer in the PMSG group. The fertilization rate within peforelin trials 1 and 3 halved as the amount of injected hormone decreased. Considerable fluctuations in fertility rates were observed in three separate experiments with equal amounts of PMSG. The fertility rate was reduced by more than half compared to trial 1 in the PMSG cohort (Table 3).

In parallel with the evaluation of the oocytes retrieved from the fallopian tube and differentiation steps after IVF, the ovaries of the mice were subjected to histological examination (Figure 1). The ovaries were completely sectioned and the slides were examined at fixed intervals and evaluated according to morphological criteria (Table 1).

**Figure 2:**
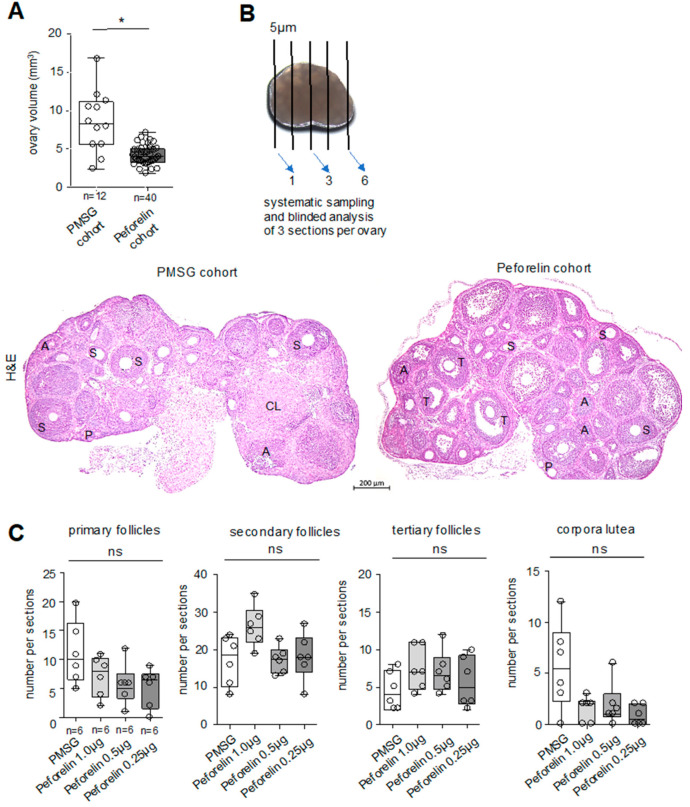
Evaluation of ovary morphology and follicular development in PMSG- and peforelin-treated animals. (**A**) Ovary volume of PMSG and peforelin cohorts. (**B**) Workflow of ovary sectioning and representative H&E-stained section of PMSG- and peforelin-treated animals. (**C**) Quantification of follicular differentiation stages and corpora lutea in all trails. Significance level *p* < 0.05, ns = not significant, n = number of animals. Section thickness 5 µm (**B**); scale bar 200 µm (**B**). * significant differences inbetween cohorts.

In order to study the influence of hormone administration on the ovary and the follicular differentiation stages up to the corpus luteum, the ovaries were removed immediately after euthanasia. Stimulation with PMSG showed a significant increase in ovarian volume compared to the peforelin cohort (Figure 2A). To further investigate the effects of hormone administration and the different concentrations on ovarian morphology, serial sections of the ovaries were prepared and histologically analyzed (Figure 2B). Evaluation of follicular differentiation stages showed a clear, but not significant, increase in the number of primary follicles in the PMSG cohort compared with the peforelin-treated group (Figure 2C). The number of secondary follicles was increased in peforelin trial 1 (1.0 µg cohort), whereas trials 2 and 3 did not differ from the PMSG cohort. In addition, the number of tertiary follicular stages showed no significant difference between the PMSG and peforelin cohorts (Figure 2C). The corpus luteum was identified in all groups, but more structures were visible in the PMSG cohort than in the three trials with peforelin (Figure 2C).

**Figure 3:**
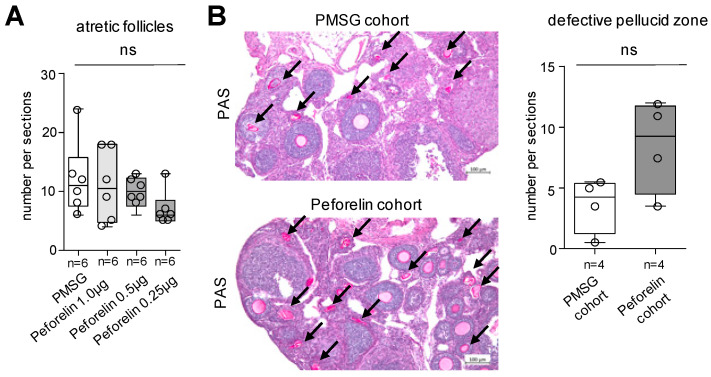
Quantification of atretic follicles in PMSG- and peforelin-treated animals. (**A**) Quantification of atretic follicles in PMSG and peforelin trials. (**B**) Representative PAS-stained section and quantification of defective zona pellucida (arrows) in PMSG and peforelin cohorts. n = number of animals. Significance level *p* < 0.05, ns = not significant, scale bar 100 µm (**B**).

Follicular differentiation stages were also assessed for the presence of cellular signs for apoptosis. There were no significant differences in the number of atretic follicles in the PMSG and peforelin cohorts. A slight trend towards a lower number of apoptotic cells was observed in the peforelin cohorts (Figure 3A). To investigate this aspect using a further criterion, we examined the morphology of the zona pellucida (ZP) in PAS-stained sections. More structures with defective ZP were identified in the peforelin cohorts compared to the PMSG controls (Figure 3B).

## 4. Discussion

PMSG, a hormone derived from the blood of pregnant mares, plays a central role in animal breeding and production [9]. In laboratory animal science, PMSG is used for superovulation in the context of reproductive biology interventions in various species including mice, rats, and hamsters [2,3,4,5,6,7,8,9,10,11,12,13,14,15,16,17,18]. The method of obtaining PMSG has come under increasing ethical scrutiny due to the significant stress and potential suffering it can cause to the horses [5]. Studies have shown that blood sampling from pregnant mares can cause both physical and psychological stress [19]. These ethical concerns have led to intense debate on the need to develop alternatives, particularly synthetic/recombinant derivatives [5,9,12]. The development and implementation of such alternatives could make a significant contribution to improving animal welfare by reducing the reliance on PMSG and the associated ethical concerns.

In veterinary practice, GnRH analogs, usually administered transvaginally, are already in use in cattle, pigs, and rabbits [14,20,21]. Recently, GnRH downstream hormones such as recombinant FSH have also been evaluated in ruminants [10]. One of the most promising alternatives to PMSG is peforelin, a synthetic gonadotropin agonist that has similar effects to PMSG after injection and was first used in pig reproduction [14,15]. To evaluate the effect of peforelin as an alternative to PMSG in laboratory animals, we compared three different concentrations of peforelin to PMSG to induce superovulation and follicular differentiation in the commonly used mouse line C57BL/6J.

The protocol we used to induce superovulation is consistent with studies in C57BL/6 inbred mice which have shown that high numbers of oocytes can be produced by the use of 5 IU to 7.5 IU PMSG followed by an injection of hCG [18,22,23]. Whilst there are various protocols for inducing superovulation in mice, there are none for the use of peforelin in laboratory animals. We therefore followed our standard protocol using 5 IU PMSG and the time intervals depicted in Figure 1. In parallel, we tested peforelin at three different concentrations from 1.0 µg, 0.5 µg, to 0.25 µg (trials 1–3).

We first analyzed the dissected oviducts and the oocytes they contained and their ability to differentiate to the blastocyte stage after in vitro fertilization. Overall, the data obtained showed that peforelin, at all concentrations tested, was less effective than the PMSG control in stimulating oocyte development in C75BL/6J mice. The study by Gates and Bozarth, as well as other reports, noted that the response to superovulation in mice can vary significantly depending on factors such as dose, age, and strain [1,22,24]. Here, we tested 3–4-week-old C57BL/6J mice and obtained high numbers of oocytes with our protocol; similar results have been obtained in comparable studies [24,25]. Of note, the use of immature oocyte donors in assisted reproduction has been reported to be suitable for producing high numbers of oocytes with a shortened developmental interval [26]. Importantly, taking into account the lower number of oocytes produced by peforelin, the cellular differentiation into blastocysts after in vitro fertilization was not altered, especially when using 1.0 and 0.5 µg concentrations. This finding indicates that the oocyte quality and fertilization potential in the peforelin cohort are generally sufficient. The fertilization rate in C57BL/6J mice is reported to be around 80% [27]. Here, we obtained lower percentages of fertilization rates in all trials evaluated, which has already been reported for different concentrations of GnRH analogs in mice [28]. The lower fertilization rates could be due to differences in sperm quality or different IVF protocols used. Interestingly, when the same cryopreserved sperm and protocol were applied to peforelin-produced oocytes, the cohort showed consistently higher fertilization rates compared to oocytes from the PMSG cohort. Results from the study by Vangroenweghe et al. showed an increase in the farrowing rate after stimulation with peforelin. The greater farrowing success may indicate that, similar to the data we obtained in mice, the fertilization ability and further differentiation properties of oocytes obtained from peforelin administration are good [15].

We also analyzed the effect of peforelin on ovarian morphology. We observed a significant increase in ovarian volume in the PMSG cohort, suggesting a high degree of ovarian stimulation compared to the peforelin cohort. Indeed, gonadotropins stimulate ovarian remodeling [29]. In this regard, ovarian weight may be mainly influenced by increased follicular differentiation and accelerated luteinization [28]. We observed higher numbers of primary follicles and corpora lutea in the PMSG-treated cohort, which is consistent with previous reports showing that gonadotropins induce rapid follicular differentiation and corpora lutea formation [3,28]. This is supported by data from gilts and pigs, on whom peforelin has already been tested and has led to an increase in follicle size [14]. Additionally, the number of secondary and tertiary follicles was slightly different between peforelin- and PMSG-treated mice. It is interesting to note that mice in the peforelin cohort had a higher number of secondary follicles. Wang et al. have shown that high doses, but not low doses, of gonadotropins lead to increased numbers of secondary follicles, especially when the time interval before the hCG stimulation is shortened [28]. This also suggests that peforelin at doses of 1.0–0.5 µg is indeed able to induce significant ovarian stimulation and follicular differentiation, whereas the time window of hCG administration seems to be the more critical factor for ovulation induction.

In support of the benefical effects of peforelin on ovarian remodeling, there were no significant changes in the number of atretic follicles in 1.0 µg and 0.5 µg treated mice compared to PMSG-treated mice. Follicular atresia is a common finding in the process of differentiation, especially in the case of over-tuning with administered gonadotropins [30]. Based on the quantification of atretic secondary and tertiary follicles by the presence of pyknotic granulosa cells, which in turn can lead to a high number of false-positive results [31], we also examined the morphology of the zona pellucida (ZP) using PAS staining. Detection of the highly stained ZP remnants, which appear as wavy, contracted structures, showed that many more follicles perished in the peforelin group than in the PMSG cohort. This finding is consistent with the benefical effect of peforelin on ovarian morphology, particularly in inducing follicular differentiation. We hypothesize that the almost identical number of differentiating follicles and the high number of atretic follicles indicate a delayed follicular maturation compared to PMSG-treated animals.

As Edgar et al. have shown that excessive administration of gonadotropins has a rather negative effect on oocyte differentiation [32], we suggest a concentration of 0.5 µg peforelin for further studies. At this dose, good results were obtained in our study in terms of both oocyte morphology and yield. Shortening the time between gonadotropin stimulation and hCG administration could have an effect on the number of oocytes obtained, or pretreatments with GnRH prior to peforelin–hCG administration, adjustments which have been used on mice [22,33], may overcome the limitations of peforelin compared to PMSG. We do not expect that increasing the time window between hCG administration and oocyte collection will be beneficial in C57BL/6J mice, as prolonged time windows will lead to reduced fertilization rates in this strain [34]. This hypothesis needs to be further investigated in order to establish peforelin as an alternative to PMSG in the context of the 3Rs principle.

## 5. Conclusions

These results suggest that peforelin is able to stimulate ovarian remodeling, follicular differentiation, and oocyte development with high rates of fertilization. Unless the number of oocytes per animal was low for the three different doses tested in our study, we propose that from an animal welfare perspective, peforelin is a promising alternative to PMSG for specific applications in laboratory animal science. Clearly, further research is needed to establish appropriate protocols for the use of peforelin in laboratory mice.

## Figures and Tables

**Table 1:** Histological classification of the differentiation stages in the ovary.

Primary follicles	The oocyte was surrounded by one layer of cuboidal granulosa cells (GCs)
Secondary follicles	Showed at least two layers of GCs surrounding the oocyte but no antral cavity
Tertiary follicles	Antral follicles were counted when more than two layers of healthy GCs around the oocyte were visible and an antrum was present
Atretic follicles	In general, if more than three pyknotic nuclei within secondary or tertiary follicles were observed, atresia was assumed
Corpus luteum	Large, pale-stained cells originate from granulosa cells of the follicle

**Table 2:** Comparison of three different concentrations of peforelin to PMSG control groups.

	Trial 1		Trial 2		Trial 3		
	1.00 µg Peforelin	5 IU PMSG	0.50 µg Peforelin	5 IU PMSG	0.25 µg Peforelin	5 IU PMSG	*p*
N	14	110	31 *	95	20 *	67	<0.05
2C	14	73	26 *	73	10 *	18	<0.05
4C	14	63	22 *	58	5 *	18	<0.05
Mo.	11	63	22 *	58	4 *	15	<0.05
Bl.	11	55	20 *	58	4 *	15	<0.05

N = total number of cells, 2C = number of two-cell stages, 4C = number of four-cell stages, Mo. = number of morula complexes, Bl. = number of blastocytes. Significance level *p*, * statistically significant differences between trials.

**Table 3:** Fertilization rates of peforelin and PMSG cohorts.

	Peforelin (0.25, 0.5, 1.0 µg) Fertilization Rate	PMSG Control (5 IU) Fertilization Rate
Trial 1: 1.00 µg Peforelin	100%	66%
Trial 2: 0.50 µg Peforelin	83%	76%
Trial 3: 0.25 µg Peforelin	50%	26%

## Data Availability

All data are provided in the manuscript.
